# Comparison of ingesting a food bar containing whey protein and isomalto-oligosaccharides to carbohydrate on performance and recovery from an acute bout of resistance-exercise and sprint conditioning: an open label, randomized, counterbalanced, crossover pilot study

**DOI:** 10.1186/s12970-019-0301-z

**Published:** 2019-08-13

**Authors:** Tyler J. Grubic, Ryan J. Sowinski, Ben E. Nevares, Victoria M. Jenkins, Susannah L. Williamson, Aimee G. Reyes, Christopher Rasmussen, Mike Greenwood, Peter S. Murano, Conrad P. Earnest, Richard B. Kreider

**Affiliations:** 1 Exercise & Sport Nutrition Lab, Human Clinical Research Facility, Department of Health & Kinesiology, Texas A&M University, College Station, TX 77843-4243 USA; 2 Department of Nutrition and Food Sciences, Texas A&M University, College Station, TX 77843 USA

**Keywords:** Energy bars, Glycemic index, Glycemic load, Nutrient timing

## Abstract

**Background:**

We previously reported that consuming a food bar (FB) containing whey protein and the plant fiber isomalto-oligosaccharides [IMO] had a lower glycemic (GI) but similar insulinemic response as a high GI carbohydrate. Therefore, we hypothesized that ingestion of this FB before, during, and following intense exercise would better maintain glucose homeostasis and performance while hastening recovery in comparison to the common practice of ingesting carbohydrate alone.

**Methods:**

Twelve resistance-trained males participated in an open label, randomized, counterbalanced, crossover trial with a 7-d washout period. Participants consumed a carbohydrate matched dextrose comparitor (CHO) or a FB containing 20 g of whey, 25 g of IMO, and 7 g of fat 30-min before, mid-way, and following intense exercise. Participants performed 11 resistance-exercises (3 sets of 10 repetitions at 70% of 1RM) followed by agility and sprint conditioning drills for time. Participants donated blood to assess catabolic and inflammatory markers, performed isokinetic strength tests, and rated perceptions of muscle soreness, hypoglycemia before, and following exercise and after 48 h of recovery. Data were analyzed using general linear models (GLM) for repeated measures and mean changes from baseline with 95% confidence intervals (CI) with a one-way analysis of variance. Data are reported as mean change from baseline with 95% CI.

**Results:**

GLM analysis demonstrated that blood glucose was significantly higher 30-min post-ingestion for CHO (3.1 [2.0, 4.3 mmol/L,] and FB (0.8 [0.2, 1.5, mmol/L, *p* = 0.001) while the post-exercise ratio of insulin to glucose was greater with FB (CHO 0.04 [0.00, 0.08], FB 0.11 [0.07, 0.15], *p* = 0.013, η^2^ = 0.25). GLM analysis revealed no significant interaction effects between treatments in lifting volume of each resistance-exercise or total lifting volume. However, analysis of mean changes from baseline with 95% CI’s revealed that leg press lifting volume (CHO -130.79 [− 235.02, − 26.55]; FB -7.94 [− 112.17, 96.30] kg, *p* = 0.09, η^2^ = 0.12) and total lifting volume (CHO -198.26 [− 320.1, − 76.4], FB -81.7 [− 203.6, 40.1] kg, *p* = 0.175, η^2^ = 0.08) from set 1 to 3 was significantly reduced for CHO, but not for the FB. No significant interaction effects were observed in ratings of muscle soreness. However, mean change analysis revealed that ratings of soreness of the distal vastus medialis significantly increased from baseline with CHO while being unchanged with FB (CHO 1.88 [0.60, 3.17]; FB 0.29 [− 0.99, 1.57] cm, *p* = 0.083, η^2^ = 0.13). No significant GLM interaction or mean change analysis effects were seen between treatments in sprint performance, isokinetic strength, markers of catabolism, stress and sex hormones, or inflammatory markers.

**Conclusion:**

Pilot study results provide some evidence that ingestion of this FB can positively affect glucose homeostasis, help maintain workout performance, and lessen perceptions of muscle soreness.

**Trial registration:**

clinicaltrials.gov, #NCT03704337. Retrospectively registered 12, July 2018.

**Electronic supplementary material:**

The online version of this article (10.1186/s12970-019-0301-z) contains supplementary material, which is available to authorized users.

## Background

Ingestion of carbohydrate and protein before, during, and/or following exercise has been reported to enhance energy substrate availability, sustain exercise performance, and promote recovery [1, 2]. For this reason, active individuals often ingest energy drinks, gels and/or bars before, during, and/or following exercise [1–4]. However, most commercially available energy drinks, gels, and bars have a relatively high glycemic index (GI) and therefore may not be not suitable for individuals who are glucose intolerant, diabetic, or susceptible to hypoglycemia during exercise [1, 2, 4–6]. There has also been significant interest in identifying how carbohydrate, protein, and/or amino acids consumption influence exercise capacity and/or performance. Research has shown that different types of carbohydrate and protein can have varying effects on substrate availability, exercise metabolism, performance, and/or recovery. For example, we previously reported that ingestion of moderate to low GI carbohydrate gel during prolonged cycling maintained blood glucose and insulin levels to a greater degree than a higher GI gel [6]. Additionally, adding different types of carbohydrate with low to high GI’s to whey protein had differential effects on glucose and insulin responses following intense resistance-exercise [5]. Based on this type of research, it has been recommended that athletes consume low to moderate GI carbohydrate before and during exercise [1, 2]. Moreover, consuming whey protein and/or essential amino acids before, during, and/or following intense exercise can enhance protein synthesis [1, 2].

Isomalto-oligosaccharides (IMO) is a prebiotic high fiber, low calorie source of carbohydrate that has been used as a functional food and prebiotic fiber sweetener in Asia for over 3 decades [7–11]. Basic animal studies indicate that IMO’s serve as a soluble dietary fiber and can stimulate activity of the probiotic gut flora, improve gut function, and help manage cholesterol in animals fed on a high fat diet [7, 10, 12–14]. Given the interest in developing food and energy bars that provide quality protein with a low to moderate glycemic profile, we previously reported that ingesting a whey protein energy bar with IMO as the source of carbohydrate had a GI of 34 and a glycemic load of 8.5 [15]. Additionally, we reported that ingesting this energy bar increased insulin to a greater degree while maintaining blood glucose compared to a dextrose control [15]. Theoretically, ingestion of this food bar before, during, and/or following intense exercise could maintain blood glucose and increase insulin levels during exercise, lessen the catabolic effects of intense exercise, reduce the inflammatory response to exercise, and/or hasten recovery.

The purpose of this study was to determine if ingesting this low-glycemic food bar before, during, and following an intense resistance and sprint-conditioning workout, as would typically be used in an off-season collegiate strength and conditioning program for strength/power athletes, would affect glucose homeostasis, exercise performance and/or recovery. The primary outcome, measure was glucose homeostasis during and following exercise. Secondary outcome measures included assessment of performance, ratings of muscle soreness, markers of catabolism and inflammation, and subjective ratings of appetite, hypoglycemia, and readiness to perform. We hypothesized that ingestion of the FB studied would better maintain glucose homeostasis, better maintain exercise capacity during intense training, and hasten recovery in comparison to the common practice of ingesting carbohydrate alone.

## Methods

### Experimental design

This pilot study was conducted at a university research setting with approval by an Institutional Review Board (IRB2017–0602) in compliance with the Declaration of Helsinki standards for ethical principles regarding human participant research. The study was registered retrospectively with clinicatrials.gov (# NCT03704337). This pilot study was conducted in a randomized, counter-balanced, crossover, and open label manner. The independent variable was nutrient intake. The primary outcome measure was glucose homeostasis as determined by assessing glucose and insulin responses. Secondary outcome measures included assessment of performance as determined by assessing resistance-exercise lifting volume, agility and sprint performance, and isokinetic strength; and, recovery as determined by assessing ratings of muscle soreness; markers of catabolism, stress, and inflammation; and, ratings of readiness to perform. Additionally, dietary energy and macronutrient, subjective ratings of symptoms of hypoglycemia and subjective ratings of appetite and eating satisfaction were assessed.

### Participants

**Fig. 1:**
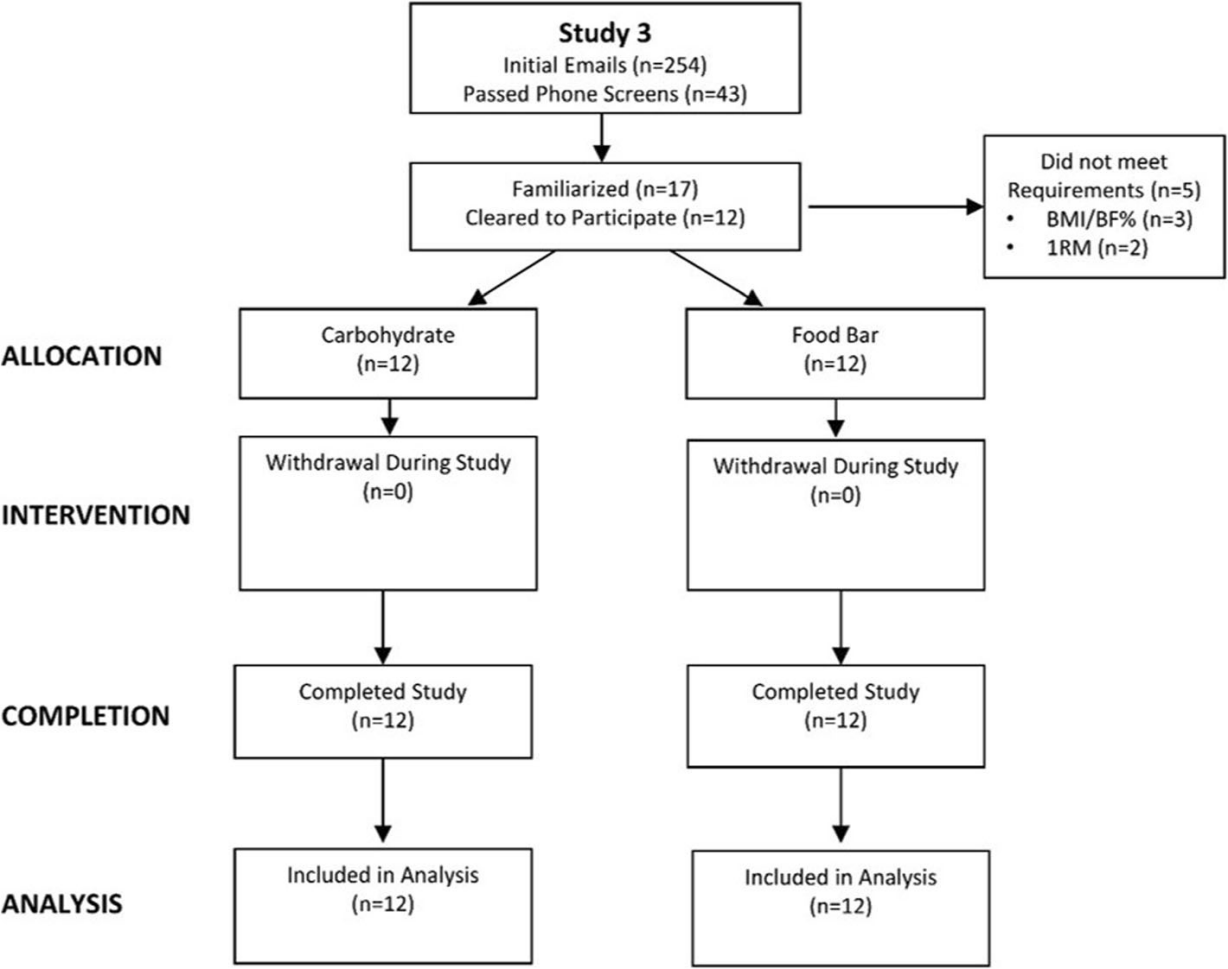
Consolidated Standards of Reporting Trials (CONSORT) diagram

Twelve highly-trained men between the ages 18–35 years with a body fat percentage (BF%) less than 25% or body mass index (BMI) less than 25 kg/m^2^, were recruited to participate in this study. Participants were required to: 1.) have the capability to bench press their body weight and barbell squat at least 1.5 times their body weight; 2.) have been engaged in a resistance training program involving upper and lower body exercises for the last year; and, 3.) involved in sprint conditioning training for the last 6 months. Pre-screening questionnaires and interviews revealed that all of the participants were former high school and/or college athletes who participated in competitive intramural sports on campus and/or routinely performed training programs involving heavy resistance-exercise and sprint conditioning similar to the exercise bout used in this study. Since all athletes perform strength and conditioning programs like the one used in this study regardless of sport and/or position and the participants served as their own control, we did not limit participants to specific sport backgrounds and/or positions within a given sport. Individuals who expressed interest in participating in the study were screened by phone to determine if they met initial eligibility criteria to participate in this study. Qualified individuals were invited to attend a familiarization session in which participants received a written and verbal explanation of the study design, testing procedures, and read and signed informed consent statements. Those giving consent completed personal, training, and medical histories and had a physical examination by a research assistant. The research coordinator reviewed medical history forms, physical examination measurements, and determined eligibility to participate. Participants were excluded from the study if they reported: 1) any uncontrolled metabolic disorders or cardiovascular disorder, including heart disease, a history of hypertension, diabetes, thyroid disease, hypogonadism; 2) hepatorenal, musculoskeletal, autoimmune, or neurological disease; 3) they were currently taking prescribed medication or dietary supplements for thyroid, hyperlipidemia, hypoglycemia, anti-hypertensive, anti-inflammatory, weight loss (e.g. thermogenic compounds) within 3 months before the start of this study; 4) had any known allergies to some of the nutrients contained in the food bar (i.e., almonds, milk, soy, peanuts, tree nuts, egg, and wheat); 5) did not meet BF% or BMI criteria; or, 6) did not meet bench press and/or squat one repetition maximum (1RM) criteria. Figure 1 presents a Consolidated Standards of Reporting Trials (CONSORT) diagram for the study. A total of 43 individuals passed phone screens, 17 participants gave consent to participate in the study and underwent familiarization, 12 individuals met all screening criteria and were allocated to start the study and 12 participants completed the study.

### Nutritional intervention

In a counterbalanced, crossover, and open label manner; participants ingested 25 g of dextrose gel (*Valeant Pharmaceuticals North America LLC, Bridgewater, NJ, USA*) which served as a carbohydrate-matched comparator (CHO) or a commercially-available food bar (FB, *FitJoy™, Nutrabolt, Bryan TX*) containing 20 g of a whey protein, 25 g of carbohydrate as IMO plant fiber (*VitaFiber™, BioNutra North America, Inc. Edmonton, Alberta, Canada*) consisting of 13 g fiber and 4 g of sugar, and 7 g of fat (1.5 g saturated fat) before, during, and following intense exercise. Supplements were randomly assigned in an alternating fashion to counterbalance the design. Participants were informed that the purpose of the study was to compare the effects of two common nutritional strategies on exercise performance and recovery. Participants were given as much time as needed to ingest the nutrients which typically lasted 3–5 min. One FB contained 220 cal while the PL contained 100 cal of carbohydrate. The rationale in using a carbohydrate matched dextrose gel rather than a iso-caloric amount of carbohydrate was that: 1.) Athletes typically ingest carbohydrate drinks and/or gels before and during exercise so efficacy of the FB would need to be established in comparison to common practice; 2.) The amount of carbohydrate used was consistent with recommendations of the amount of carbohydrate per hour athletes should consume (i.e., 30–60 g/h or carbohydrate) [1, 2]; 3.); 3.) Providing an iso-caloric amount of carbohydrate gel to match the energy intake of the FB (i.e., 3 × 55 g per servings over a 1.25 h period of training) would have likely promoted hypoglycemia and impaired exercise performance; and, 4.) Costs of manufacturing an energy bar containing all nutrients with a different source of carbohydrate for this initial exploratory pilot study was cost prohibitive. After a 7-day washout period, participants repeated the experiment while ingesting the remaining nutritional intervention.

### Testing sequence

**Fig. 2:**
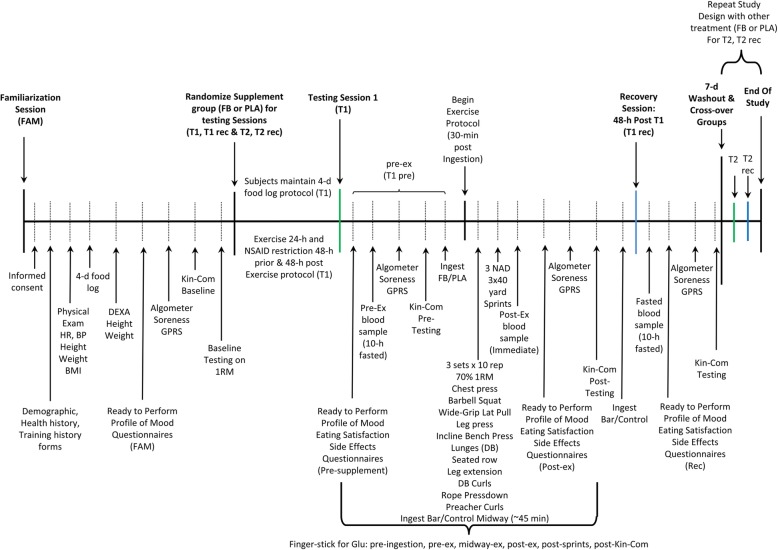
Timeline for testing. NSAID = non-steroidal anti-inflammatory drugs, FB = food bar, CHO = carbohydrate, 1RM = one repetition maximum, BG = blood glucose, NAD = Nebraska Agility Drill

Figure 2 presents the general experimental design employed in this study. Participants were instructed to refrain from non-steroidal anti-inflammatory drug (NSAID) and pain relief medication for 48 h, exercise for 24 h, and fast for 10 h before reporting to the lab for testing. Once arriving at the lab participants completed appetite and food satisfaction, symptoms of hypoglycemia, and readiness to perform questionnaires; and, donated a fasting blood sample. Baseline ratings of pain to a standard amount of pressure applied to several locations on the thigh, isokinetic muscular strength and endurance measurements, and arterialized-venous glucose measurements from a finger were then obtained. Participants then ingested their assigned nutrient (CHO or FB) and rested passively for 30 min. Participants then completed a rigorous resistance-training exercise protocol consisting of 11 total upper and lower body exercises. Midway through the exercise session, participants ingested another serving of the CHO or FB. After the resistance-exercise was completed, participants performed three 40-yard (FYD) and three repeated Nebraska Agility Drills (NAD) utilizing a 1:4 work to rest ratio. Arterialized-venous samples were also taken immediately before exercise, midway during resistance-exercise, following resistance-exercise, following performing the sprints, and following isokinetic testing. After completing the exercise bout, participants completed questionnaires, donated a venous blood sample, rated pain to standard pressure applied to the thigh, and performed isokinetic tests. Participants consumed a final serving of CHO or FB before leaving the lab and were instructed not to eat any additional food for another 2 h in order standardize the amount of time the nutritional interventions would have on muscle protein synthesis after exercise before additional nutrients were consumed. Participants refrained from exercise and NSAID or pain relief medication during the 48-h recovery period. Participants then reported to the lab 2 days later after fasting for 10 h. Participants then donated a venous blood sample, rated pain to a standard amount of pressure applied to the thigh, and performed isokinetic testing. Participants observed a 7-day washout period and then repeated the experiment in a crossover manner while ingesting the alternative nutrient. Participants were asked to follow a similar training and diet pattern that they followed leading up to performing their first treatment intervention session.

## Procedures

### Demographics

Body weight and height was determined on a Healthometer Professional Scale model 500KL (Pelstar LLC, Alsip, IL, USA). Heart rate was taken at the radial artery and systolic and diastolic blood pressure was measured using standard procedures [16]**.** Body composition was determined with a Hologic Discovery W Dual-Energy X-ray Absorptiometer (DXA; Hologic Inc., Waltham, MA, USA) equipped with APEX Software (APEX Corporation Software, Pittsburg, PA, USA)**.** Test-retest reliability studies performed with this DXA machine have previously yielded mean coefficients of variation (CV) for bone mineral content and lean mass of 0.31–0.45% with a mean intra-class correlation of 0.985 [17].

### Dietary assessment

Participants were instructed to record all food and beverage intakes each week that they were involved in the study protocol on 4-day dietary food logs (3 weekdays, 1 weekend day), which is reflective of their average dietary intake on normal days. Food records were entered and analyzed with Food Processor Nutrition Analysis Software Version 11.2.285 (ESHA Nutrition Research, Salem, OR) and analyzed for average energy and macronutrients by study researchers [18].

### Resistance exercise protocol

During the familiarization testing session participants followed a protocol to determine 1RM for chest press, barbell squat, wide-grip latissimus dorsi (lat) pull, leg press, incline bench press, dumbbell lunges, seated row, leg extension, dumbbell curls, triceps rope press-down, and biceps curls [5]. For exercises in which 1RM was exceeded by available weights, the Epley formula was used to predict the 1RM based on the number of repetitions performed at a given weight [19]. Rest periods between participants was not limited during 1RM determination so that the participants had sufficient opportunity to reach their true maximum weight, however participants were encouraged to try to reach their 1RM within 3–5 sets of their warmup set in agreement with standard testing protocols [20]. During the resistance exercise protocol, each participant performed three sets of 10 repetitions with approximately 70% of their 1RM for each of the 11 total exercises (i.e., chest press, barbell squat, wide-grip latissimus lat pull, leg press, incline bench press, lunges, seated row, leg extension, dumbbell curls, triceps press-down, and biceps bar curls) [5]. Each set was followed by a 2-min rest period. All lifting was performed under the supervision of researchers and a certified strength and conditioning coach. If a participant could not complete the full 10 repetitions at the 70% 1RM load, the weight was immediately reduced so that the 10 repetitions could be completed. The weight and the number of repetitions was recorded by researchers on each participant’s workout card immediately following each completed set, so that total lifting volume could later be calculated. The resistance-training session lasted approximately 1.25 h. Test-to-test reliability for total lifting volume revealed a mean CV of 0.16 with an overall mean intraclass correlation of 0.996.

### Conditioning drills

Directly following the resistance-exercise protocol, each participant performed three 40-yard sprint trials separated by about 20-s of rest in between, to implement a 1:4 work to rest interval ratio. When ready, the participant lined up at the starting line and was instructed to sprint as fast as they could all the way through the finish line. Participants were also instructed to start in a static position, and had the option to start in a three point stance or standing, but had to maintain the same starting position for each time-trial. The recorded time for the 40-yard dash began on the participant’s first motion forward and ended once the participant crossed the finish line at 40-yards [21, 22]. The test was performed on the same gym floor for each participant with lines denoting start and stop points. Test-to-test reliability for the 40-yard dash sprint times revealed a mean CV of 0.06 with an overall mean intraclass correlation of 0.916. Participants then performed three NAD agility tests. The NAD is designed to test agility and change of direction skills [23]. The test is set up using four cones. Two cones are set up in line with one another five yards apart. One set of cones is offset by one yard on a line five yards apart from the first set of cones. Participants are asked to sprint five yards to the cone on the next line, change direction and sprint back to the next cone on the start line, change direction and sprint to the last cone on the opposite line. Timing began on the participant’s first motion forward and ended once the participant crossed the last cone. Each participant completed three trials of this drill for time, implementing a 1:4 work to rest ratio. The conditioning drill session lasted approximately 0.25 h. Test-to-test reliability for the NAD sprint times revealed a mean CV of 0.08 with an overall mean intraclass correlation of 0.792. Total exercise time to complete the resistance training and sprint conditioning drills was approximately a 1.5 h.

### Muscle soreness assessment

A Commander algometer (JTECH Medical, Salt Lake City, UT, USA) was used to apply a standardized amount of pressure (50 N) to the vastus lateralis at the distal 25% (DVL) and 50% midpoint (MVL) of the distance between the superior border of the patella and the greater trochanter of the femur and to the vastus medalis (VM) at 25% of the distance between the aforementioned landmarks. The three sites were marked with permanent ink to standardize the location of assessment. Participants were asked to sit with both legs straight on a bench while the algometer measurements were taken. Pressure was applied to each site for 3-s as previously described [24]. Participants were asked to rate their perception of muscle soreness using a graded visual analog scale (GPRS) at each site. The GPRS consisted of a straight horizontal-line with no hash-markings and only wording beneath the line. From left-to-right, the line read “no pain, dull ache, slight pain, more slight pain, painful, very painful, and unbearable pain”. Participants were instructed to scribe one clear mark bisecting the line, which represented their pain level the best for each of the three pressure application sites. A ruler was used to measure the participant’s mark from the left-to-right in cm and was recorded in the data as such numerical value. Testing order (i.e., VM, DVL, MVL) was standardized across testing sessions. Participants recorded their perceived level of soreness on the GPRS evaluation line before moving onto the next site. Test to test variability of performing this test yielded mean CV values ranging from 0.40 to 1.10 with mean a intraclass correlation of 0.90 [24].

### Isokinetic assessment

Participants performed a maximum voluntary contraction (MVC) isokinetic knee extension and flexion protocol at a speed of 60 degrees/sec on their dominant leg using the Kin-Com 125AP Isokinetic Dynamometer (Chattanooga-DJO Global Inc., Vista, CA, USA). Body and knee positioning were pre-determined during a familiarization session, and recorded using standard procedures to ensure testing was repeatable and to decrease any between-testing variability for all isokinetic tests performed throughout the testing duration. Each participant went through a warm up protocol before testing by performing three sets of five repetitions of knee extension and flexion at approximately 50% of their MVC with 1 min between sets. One minute after completing the final warm-up set, participants performed three MVC’s of knee extension and flexion [24]. Test to test variability of performing this test yielded mean CV values ranging from 0.19 to 0.21 with intraclass correlations ranging from 0.65 to 0.87 for leg extension variables and mean CV values ranging from 0.27 to 0.33 with intraclass correlations ranging from 0.77 to 0.86 for leg flexion variables.

### Blood collection and analysis

Arterialized-venous blood samples were obtained from a clean and dried finger and measured for blood glucose using an Accu-Check Aviva Plus Blood Glucose Monitoring System (Roche Diagnostics, Indianapolis, IN, USA). Additionally, approximately 20 mL of venous blood was collected in 8.5 mL BD Vacutainer® serum separation tubes (Becton, Dickinson and Company, Franklin Lakes, NJ, USA) using standard procedures [25, 26]. Samples were left at room temperature for 15 min before being centrifuged at 3500 rpm for 10 min using a refrigerated (4 °C) Thermo Scientific Heraeus MegaFuge 40R Centrifuge (Thermo Electron North America LLC, West Palm Beach, FL, USA) [27]. Serum was aliquoted into serum storage containers (Eppendorf North America, Inc., Hauppauge, NY, USA) and frozen at − 80 °C for subsequent analysis. Serum glucose and markers of catabolism were analyzed using a Cobas c111 (Roche Diagnostics, Basel, Switzerland) automated clinical chemistry analyzer. Quality control was performed daily to determine whether the system calibrated to acceptable standards using two levels of controls. Serum samples were re-analyzed if values were outside the control values or clinical normality. This analyzer has been known to be highly valid and reliable in previously published reports [25]. Test-to-test reliability assessment yielded reliability CV’s ranging between 0.4–2.4% for low control samples and 0.6–1.9% for high controls. Serum insulin, testosterone, and cortisol were analyzed using an Immulite 2000 analyzer (Siemens Healthcare GmbH, Henkest, Erlangen, Germany). Test to test reliability CV’s conducted on low and high controls ranged from 1.9–2.4% for insulin, 3.2–8.6% for cortisol, and 1.8–3.0% for testosterone. Serum inflammatory markers [interleukin (IL)-1β, IL-4, IL-6, IL-8, IL-13, tumor necrosis factor-α (TNF-α), interferon- γ (IFN-γ)] were measured using a MILLIPLEX Human High Sensitivity T-Cell Magnetic Bead Panel kit (EMD Millipore Corporation, St. Charles, MO, USA). Cytokine and chemokine measurements were assed using a Luminex MagPix instrument (Luminex Corporation, Austin, TX, USA) which requires a minimum of 50 positive beads for each human sample. This instrument has been reported to be highly reliable and valid [28–30]. Controls and all samples were run in duplicate according to standard procedures to ensure validity. The CV’s for these assays ranged between 0.02 and 1.73%.

### Questionnaires

Participants were asked to subjectively rate appetite, hunger, satisfaction from food, feelings of fullness, and amount of energy using a 0 to 10 Likert scale where 0 was none, 2.5 was low, 5 was moderate, and 7.5 was high, and 10 was severe. Test to test variability of performing this survey yielded mean CV’s ranging from 0.31 to 1.1 with mean intraclass correlations ranging from 0.42 to 0.81 for individual items on the survey. Participants were asked to rank the frequency and severity of the symptoms (i.e., hypoglycemia, dizziness, headache, fatigue, stomach upset) using the following scale: 0 (none), 1–4 (light), 5–6 (mild), 7–9 (severe), or 10 (very severe). Test to test variability of performing this survey yielded mean CV’s ranging from 1.2 to 2.6 with mean intraclass correlations ranging from 0.59 to 0.88 for individual items on the survey. Participants were also asked to rank how well they slept the night before, whether they were looking forward to the workout, how optimistic they were about their performance, how vigorous and energetic they felt, their appetite level, and amount of muscle soreness they perceived on a Readiness to Perform using the following scale: 1 (strongly disagree), 2 (disagree), 3 (neutral), 4 (agree), 5 (strongly agree). Test to test variability of performing this survey yielded mean CV’s ranging from 0.14 to 0.28 with mean intraclass correlations ranging from 0.14 to 0.76 for individual items on the survey.

### Statistical analysis

Data were analyzed using IBM® SPSS® Version 25 software (IBM Corp., Armonk, NY, USA). The sample size was based on prior research we conducted that indicated an n-size of 10 would yield a power of 0.80 on changes in glucose and insulin in response to an oral glucose challenge [5, 6]. Baseline demographic data were analyzed using descriptive statistics. Data were analyzed using a treatment (2) x time point (3 or 6) general multivariate linear model (GLM) and univariate repeated measures analysis. Wilks’ Lambda p-levels from multivariate tests are reported to describe overall time and treatment x time interaction effects of variables analyzed. Greenhouse-Geisser univariate tests were run to assess time and treatment x time interaction effects of individual variables within the multivariate model. Data were considered statistically significant when the probability of type I error was < 0.05. Least significant difference post-hoc comparisons were used to assess differences among treatments. Results with p-levels close to statistical significance (i.e., *p* > 0.05 to *p* < 0.10) are reported with partial eta-squared (η^2^) effect size where the magnitude of effect was defined as 0.01 = small, 0.06 = medium, 0.13 = large [31, 32]. Delta changes (post - pre values) and 95% confidence intervals (CI) were also calculated on the data to assess clinical significance of findings and analyzed by one-way analysis of variance (ANOVA) [33, 34]. Mean changes with 95% lower and upper CI’s completely above or below baseline were considered significantly different from baseline values [34].

## Results

### Participant characteristics

Table 1 presents participant demographics for the study. With the crossover design, there were no differences between baseline measures in demographic markers.

**Table 1:** Baseline participant demographics

Variable	Mean
Age (y)	22.0 ± 1.8
Height (m)	1.78 ± 0.06
Weight (kg)	82.8 ± 10.4
Body Fat (%)	14.2 ± 3.8
Body Mass Index (kg/m2)	26.3 ± 3.8
HR (bpm)	61.8 ± 8.5
BP Systolic (mmHg)	119.0 ± 8.8
BP Diasystolic (mmHg)	71.8 ± 5.5
Bench 1RM (kg)	103.0 ± 18.0
Squat 1RM (kg)	139.5 ± 23.6
Relative Bench Ratio	1.24 ± 0.2
Relative Squat Ratio	1.69 ± 0.2

Data are mean ± SD

### Diet analysis

Additional file 1: Table S1 presents energy and macronutrient intake data. Multivariate analysis revealed no significant overall Wilks’ Lambda for time (*p* = 0.562) or treatment x time (*p* = 0.672). Likewise, univariate analysis revealed no statistically significant interactions among treatments.

### Glycemic and Insulinemic response

Table 2 shows serum glucose and insulin data observed by treatment. Multivariate analysis revealed an overall Wilks’ Lambda time (*p* < 0.001) and treatment x time interaction (*p* = 0.007) effects. Univariate analysis revealed significant time (*p* < 0.001) but not treatment x time interactions in glucose and insulin responses. Insulin levels increased over time with no significant differences observed between treatments, although insulin was 38% higher immediately following exercise in the FB group (CHO 11.18 ± 2.69, FB 15.49 ± 2.6 uIU/mL, *p* = 0.269, η^2^ = 0.06). Univariate analysis for the insulin to glucose ratio (IGR) showed a significant effect for time (*p* < 0.001) and treatment x time (*p* = 0.008). Post-hoc analysis revealed that the IGR significantly differed between treatments after exercise.

**Table 2:** Glucose and insulin response to an oral treatment during intense exercise

Variable	Treatment	Fasted	Post-Exercise	48-h Recovery	Effect	p-Level
Glucose (mmol/L)	CHO	5.12 ± 0.48	5.05 ± 1.52	5.35 ± 0.40	Time	0.161
	FB	5.34 ± 0.40	4.81 ± 0.84	5.29 ± 0.47	Treatment x Time	0.447
Insulin (μIU/mL)	CHO	6.44 ± 3.44	11.18 ± 9.59 †	7.72 ± 3.60	Time	< 0.001
	FB	6.27 ± 3.77	15.49 ± 9.05 †	6.41 ± 3.77	Treatment x Time	0.129
	Time	6.36 ± 3.53	13.33 ± 9.38 †	7.07 ± 3.67		
IGR	CHO	0.070 ± 0.039	0.110 ± 0.072 †	0.080 ± 0.037	Time	< 0.001
	FB	0.065 ± 0.039	0.173 ± 0.085 †^	0.067 ± 0.038	Treatment x Time	0.008

Data are means ± standard deviations (SD) or standard error of the mean (SEM). A multivariate analysis revealed overall Wilks’ Lambda time (*p* < 0.001) and treatment x time (*p* = 0.007) effects. Greenhouse-Geisser univariate p-levels are presented for each variable

*CHO* Carbohydrate, *FB* Food Bar, *IGR* insulin glucose ratio

† denotes *p* < 0.05 difference from baseline. ^ represents *p* > 0.05 to *p* < 0.10 difference between CHO and FB

**Fig. 3:**
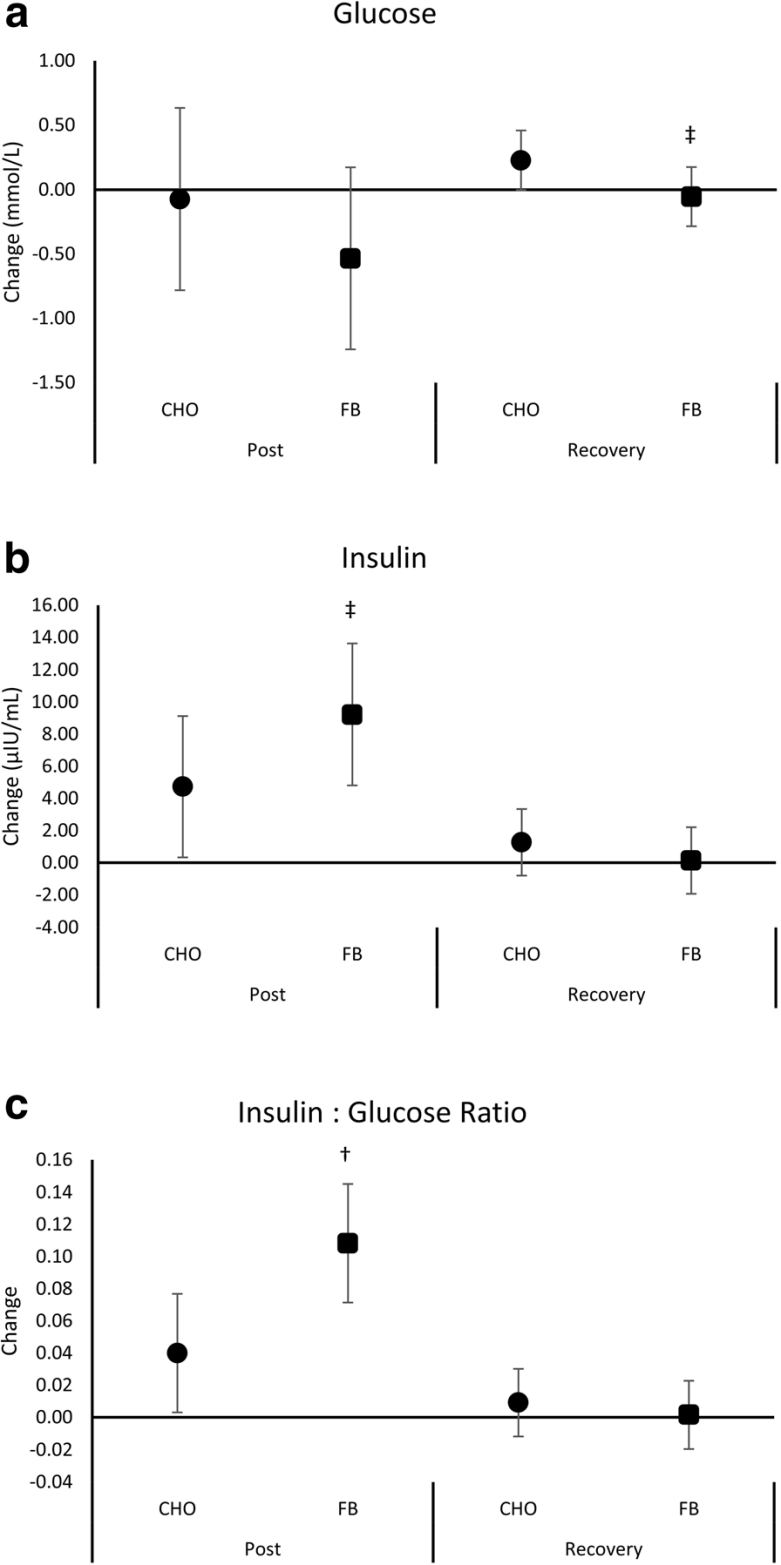
Mean changes with 95% CI in blood glucose (panel **a**), insulin (panel **b**), and the insulin to glucose ratio (panel **c**) observed in the carbohydrate (CHO) and food bar (FB) treatments. Mean changes from baseline with 95% CI’s completely above or below baseline represent a significant difference. † represents *p* < 0.05 difference between treatments. ‡ represents *p* > 0.05 to *p* < 0.10 difference between treatments

Figure 3 shows mean changes from baseline with 95% CI’s in glucose, insulin, and IGR. Glucose levels after 48-h after recovery tended to be lower in FB (CHO 0.23 [− 0.002, 0.46]; FB -0.05 [− 0.28, 0.18] mmol/L, *p* = 0.087, η^2^ = 0.13). Insulin was significantly increased above baseline values after exercise in both groups with no differences observed between treatments (CHO 4.73 [0.33, 9.14], FB 9.22 [4.82, 13.62], *p* = 0.149, η^2^ = 0.09). IGR was significantly higher in both groups post-exercise when compared to baseline, with FB being significantly higher between groups (CHO 0.04 [0.00, 0.08], FB 0.11 [0.07, 0.15], *p* = 0.013, η^2^ = 0.25). No differences were seen between groups in area under the curve.

**Fig. 4:**
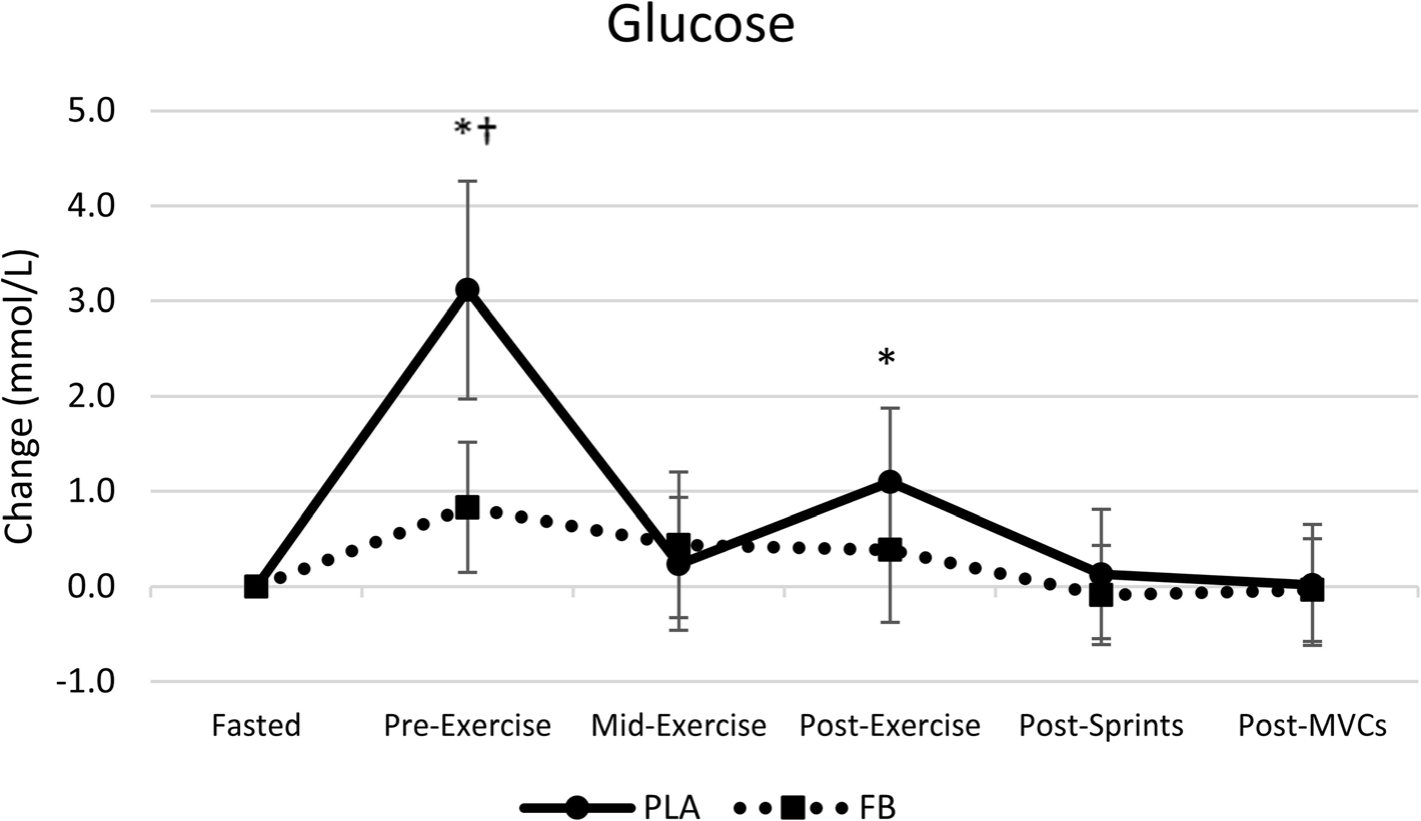
Mean changes with 95% CI in blood glucose observed in the carbohydrate (CHO) and food bar (FB) treatments. RE = resistance exercise. Mean changes from baseline with 95% CI’s completely above or below baseline represent a significant difference. * represents *p* < 0.05 difference from baseline. † represents *p* < 0.05 difference between treatments

Figure 4 presents mean changes with 95% CI’s for glucose observed during the exercise sessions. Univariate analysis revealed significant time (*p* < 0.001) and group x time interaction effects (*p* < 0.001). Blood glucose generally increased to a greater degree and for a longer period of time after ingesting the CHO. Interestingly, glucose values remained within normal values (5.3 ± 0.6 to 6.2 ± 1.0 mmol/L) throughout the entire resistance-training and sprint protocol in the FB treatment while greater variability was seen with CHO (5.3 ± 1.1 to 8.4 ± 1.6 mmol/L).

### Resistance exercise performance

Additional file 1: Table S2 presents lifting volume observed for each of the upper and lower body resistance-exercises performed in the study. Multivariate analysis revealed an overall Wilks’ Lambda time effect (*p* < 0.010) with no treatment x time interaction effect (*p* = 0.808). Univariate analysis revealed significant time effect for incline bench press (*p* < 0.002), dumbbell biceps curl (*p* = 0.001), and preacher curl (*p* = 0.032) but no significant treatment x time interaction effects in among these exercises. A

**Fig. 5:**
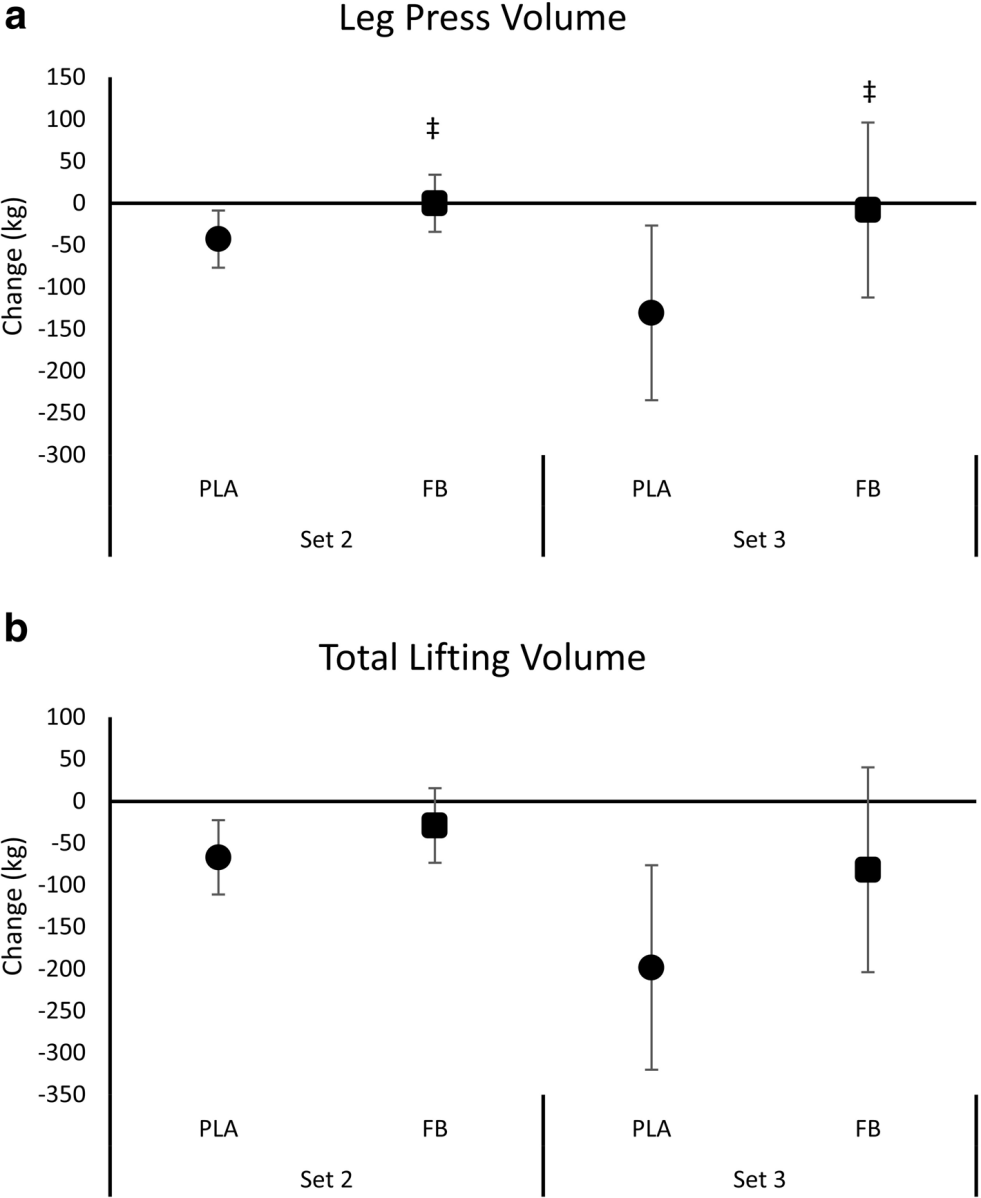
Mean changes with 95% CI in leg press volume (panel **a**) and total lifting volume (panel **b**) for the carbohydrate (CHO) and food bar (FB) treatments. Mean changes from baseline with 95% CI’s completely above or below baseline represent a significant difference. ‡ represents *p* > 0.05 to *p* < 0.10 difference between treatments

Figure 5 presents mean changes from baseline with 95% CI’s for leg press and total lifting volume. Leg press volume significantly decreased from Set 1 to Set 2 and Set 3 in the CHO treatment while participants in the FB treatment were able to maintain leg press lifting volume from Set 1 to Set 2 and Set 3. One-way ANOVA analysis revealed that leg press lifting volume tended to be lower with CHO compared to FB during Set 2 (CHO -42.71 [− 76.77, − 8.65]; FB 0.00 [− 34.06, 34.06] kg, *p* = 0.08, η^2^ = 0.13) and Set 3 (CHO -130.79 [− 235.02, − 26.55]; FB -7.94 [− 112.17, 96.30] kg, *p* = 0.09, η^2^ = 0.12) when compared to baseline. Similarly, participants maintained total lifting volume from Set 1 to Set 2 with FB treatment compared to CHO (CHO -66.9 [− 111.4, − 22.4], FB -28.9 [− 73.4, 15.6] kg, *p* = 0.224, η^2^ = 0.07) and Set 1 to Set 3 (CHO -198.26 [− 320.1, − 76.4], FB -81.7 [− 203.6, 40.1] kg, *p* = 0.175, η^2^ = 0.08). This represented a significant − 3.12% [− 5.11, − 1.14] reduction in performance from baseline in the CHO treatment compared to a non-significant − 1.28% [− 3.27, 0.71] reduction in performance from baseline in the FB treatment (*p* = 0.188, η2 = 0.08).

### Sprint performance

**Fig. 6:**
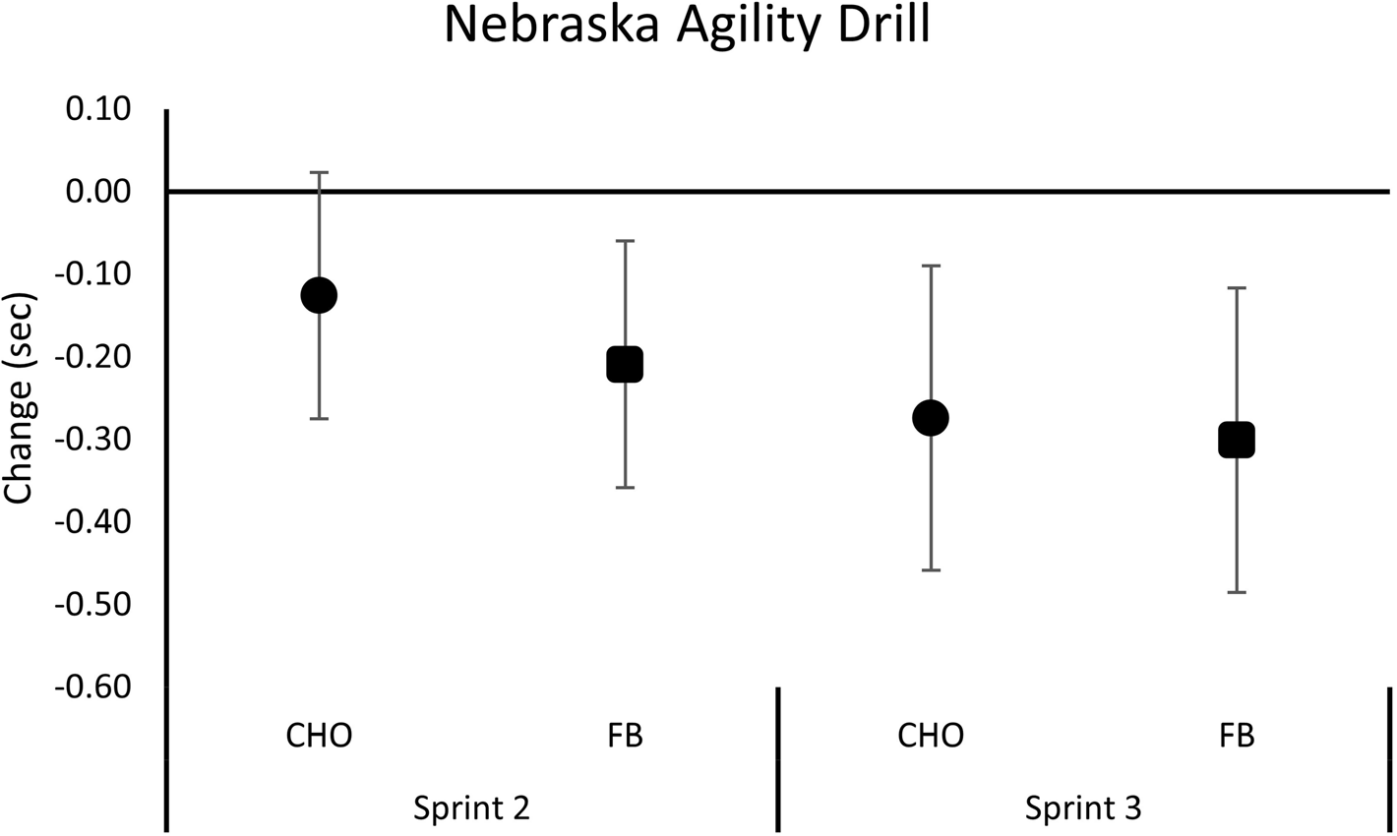
Mean changes with 95% CI in Nebraska Agility Drill performance times for the carbohydrate (CHO) and food bar (FB) treatments. Mean changes from baseline with 95% CI’s completely above or below baseline represent a significant difference

Additional file 1: Table S3 presents performance times observed for the agility and sprint tests. Multivariate analysis revealed a significant overall Wilks’ Lambda for time (*p* < 0.001) with no significant interaction effects (*p* = 0.437). Univariate analysis revealed a significant time effect for agility performance (*p* < 0.001) but not for 40-yd sprint performance (*p* = 0.252). No significant interaction effects were seen in either agility or sprint performance. Figure 6 presents mean changes from baseline with 95% CI’s for agility performance. Results revealed that agility performance in Sprint 2 were significantly faster than baseline times during the FB treatment (CHO -0.13 [− 0.28, 0.02]; FB -0.21 [− 0.36, − 0.06] sec, *p* = 0.422, η^2^ = 0.03) while both treatments were significantly faster than baseline values during sprint 3. No significant time or between group differences were observed for 40 yard dash results, although it should be noted that participants performed the first 40 yard dash Sprint − 0.15 s faster (− 2.7%) with FB treatment compared to the CHO treatment (CHO 5.50 ± 0.38; FB 5.35 ± 0.25 s, *p* = 0.251, η^2^ = 0.06).

### Isokinetic maximal voluntary contraction performance

Additional file 1: Table S4 displays the torque, force, power, and total work performed during the 3-repition isokinetic maximal voluntary extension/flexion contractions. Multivariate analysis revealed no significant overall Wilks’ Lambda time (*p* = 0.352) or treatment x time (*p* = 0.837) effects. Likewise, univariate analysis did not reveal any time or treatment x time effects for extension or flexion MVC torque, force, power, or total work. Assessment of mean changes from baseline with 95% CI’s did not reveal any significant changes from baseline or between treatments.

### Muscle soreness assessment

**Fig. 7:**
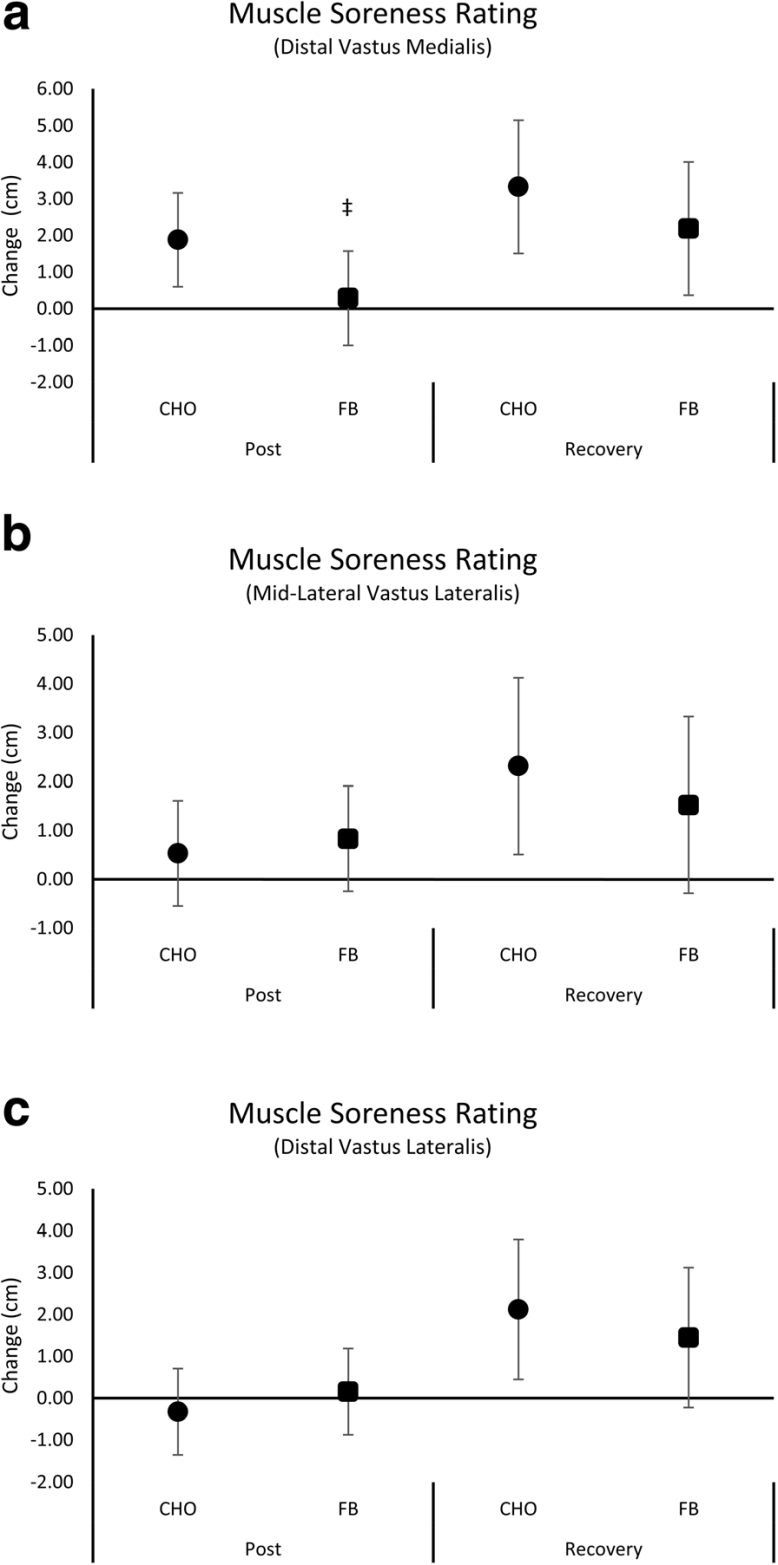
Mean changes with 95% CI in ratings of muscle soreness for the carbohydrate (CHO) and food bar (FB) treatments. Mean changes from baseline with 95% CI’s completely above or below baseline represent a significant difference. Panel **a** shows ratings for distal vastus medialis muscle soreness, Panel **b** presents ratings of muscle soreness mid-lateral vastus lateralis, and Panel **c** displays distal vastus lateral ratings of muscle soreness

Additional file 1: Table S5 presents subjective ratings of muscle soreness. Multivariate analysis revealed a significant overall Wilks’ Lambda time effect (*p* < 0.001) with no significant interaction effects (*p* = 0.538). Univariate analysis showed a significant time effect for VM (*p* < 0.001), DVL (*p* = 0.002) and MVL (*p* = 0.004) with no significant interaction effects. Figure 7 displays the mean change from baseline with 95% CI’s for ratings of muscle soreness. Ratings of VM muscle soreness after the workout were significantly increased from baseline with CHO (CHO 1.88 [0.60, 3.17]; FB 0.29 [− 0.99, 1.57] cm, *p* = 0.083, η^2^ = 0.13) while not significantly changed from baseline with FB treatment. Additionally, ratings of muscle soreness at the DVL (CHO 2.13 [0.45, 3.80]; FB 1.45 [− 0.22, 3.12] cm, *p* = 0.560, η^2^ = 0.02) and MVL (CHO 2.32 [0.51, 4.12]; FB 1.53 [− 0.28, 3.33] cm, *p* = 0.527, η^2^ = 0.02) sites remained above baseline values after 48 h recovery with CHO treatment while ratings with FB treatment were not significantly different from baseline values.

### Markers of catabolism

Additional file 1: Table S6 presents the serum markers of catabolism. Multivariate analysis revealed a significant overall Wilks’ Lambda time effect (*p* < 0.001) with no significant interaction effects (*p* = 0.360). Univariate analysis demonstrated significant effects over time for blood urea nitrogen (*p* < 0.001), creatinine (*p* < 0.001), lactate dehydrogenase (*p* < 0.001), creatine kinase (*p* = 0.038), and the blood urea nitrogen to creatinine ratio (*p* = 0.001). However, no significant univariate treatment x time interaction effects were observed.

#### Stress and sex hormones

Additional file 1: Table S7 displays the serum stress and sex hormones. Multivariate analysis revealed an overall Wilks’ Lambda time effect (*p* < 0.001) with no significant treatment x time interaction effects were observed (*p* = 0.914). Univariate analysis revealed a significant time effect for testosterone (*p* < 0.001) with no other time or interaction effects observed. Assessment of mean changes from baseline with 95% CI’s revealed that cortisol levels tended to be lower with FB treatment compared to the CHO at 48-h recovery (CHO 0.35 [− 1.18, 1.88]; FB -1.38 [− 2.90, 0.15] μg/dL, *p* = 0.111, η^2^ = 0.11). No significant differences were observed in changes in testosterone or the cortisol to testosterone ratio between treatments.

#### Inflammatory markers

Additional file 1 Table S8 presents the serum inflammatory markers analyzed. Multivariate analysis revealed a significant overall Wilks’ Lambda for time (*p* = 0.037) but not for treatment x time (*p* = 0.985). Univariate analysis revealed a time effect for IL-8 (*p* = 0.001) and TNFα (*p* = 0.044) with no significant interaction effects observed. Assessment of mean changes from baseline with 95% CI’s revealed that IL-8 was higher than baseline values following exercise with FB treatment (CHO 0.54 [− 0.07, 1.15]; FB 0.67 [0.06, 1.28] pg/mL, *p* = 0.761, η^2^ = 0.01) with no differences observed between treatments. No other differences from baseline or between treatments were observe among markers of inflammation.

### Hypoglycemia, appetite, and readiness to perform assessment

Additional file 1: Tables S9 – S11 present symptoms of hypoglycemia, appetite and eating satisfaction, and readiness to perform survey results, respectively. Multivariate analysis of responses to the eating satisfaction inventory questions revealed significant time (*p* = 0.007) with no significant interaction effects (*p* = 0.152). Univariate analysis revealed that ratings of appetite and hunger declined while feelings of fullness increased over time. A significant interaction effect was observed in feeling of fullness with food (*p* = 0.032) while ratings of hunger (*p* = 0.094) and satisfaction (*p* = 0.085) tended to differ among treatments. Assessment of mean changes from baseline with 95% CI’s revealed that hunger decreased below baseline values with FB treatment at the midway point of exercise (CHO -1.17 [− 2.65, 0.31]; FB -3.33 [− 4.81, − 1.85] *p* = 0.043, η^2^ = 0.17) and after exercise (CHO -0.75 [− 2.32, 0.82]; FB -2.42 [− 3.99, − 0.85] *p* = 0.134, η^2^ = 0.10). Ratings of appetite were significantly decreased below baseline values with FB treatment after exercise (CHO -0.67 [− 2.19, 0.85]; FB -1.92 [− 3.44, − 0.40] *p* = 0.240, η^2^ = 0.06). In terms of symptoms of hypoglycemia, a significant overall Wilks’ Lambda time effect (*p* < 0.001) was observed with no significant interaction effect (*p* = 0.269). Univariate analysis revealed a time effect for hypoglycemia (*p* = 0.001), dizziness (*p* = 0.001), fatigue (*p* < 0.001), and stomach upset (*p* = 0.004). However, no significant interaction effects were observed in ratings of symptoms of hypoglycemia, dizziness, headache, fatigue, or stomach upset. Finally, analysis of responses to the readiness to perform questionnaire revealed an overall Wilks’ Lambda time effect (*p* = 0.001) with no significant interaction effects (*p* = 0.186). Univariate analysis revealed a significant time effects for feelings of vigor and energy (*p* = 0.004), appetite (*p* = 0.035), and muscle soreness (*p* = 0.007) with no significant treatment x time interactions observed. Assessment of mean changes from baseline with 95% CI’s revealed that response to the question “*I have little muscle soreness*” were significantly decreased below baseline values with CHO treatment (CHO -1.00 [− 1.80, − 0.20]; FB -0.50 [− 1.30, 0.30] *p* = 0.368, η^2^ = 0.04) as well as after 48 h of recovery (CHO -1.00 [− 1.91, − 0.10]; FB -0.75 [− 1.66, 0.16] *p* = 0.689, η^2^ = 0.01) suggesting a greater perception of muscle soreness.

## Discussion

We previously reported that ingesting a whey protein energy bar with IMO as the source of carbohydrate had a GI of 34 and a glycemic load of 8.5 [15]. Additionally, that ingesting this energy bar increased insulin to a greater degree while maintaining blood glucose to a better degree compared to a dextrose control [15]. Theoretically, ingestion of this food bar before, during, and/or following exercise could serve as a low-glycemic source of carbohydrate and lessen the catabolic effects and inflammatory effects of intense exercise. The purpose of this pilot study was to examine the effects of ingesting a commercially available low-glycemic whey protein energy/food bar with IMO as the source of carbohydrate before, during, and following exercise affects exercise capacity and/or recovery from intense-exercise in comparison to the normally recommended practice of ingesting carbohydrate alone [1]. We hypothesized that ingestion of this whey protein food bar containing IMO would promote a low to moderate glycemic response with a similar insulin response during exercise, help athletes maintain exercise performance capacity during an intense training session, and hasten recovery. Results revealed that ingestion of this food bar promoted a more favorable glucose and insulin profile in response to intense exercise. Multivariate and univariate GLM analysis with repeated measures did not reveal significant interaction effects in performance variables, perceptions of muscle soreness, markers of catabolism, or inflammatory markers. However, there was some evidence from analysis of changes from baseline with 95% CI’s that participants ingesting the food bar were able to maintain resistance-training workloads to a greater degree during the training session as well as experienced less perception of muscle soreness during the recovery period. Therefore, there is some evidence to support our hypotheses that ingestion of this food bar may maintain glucose homeostasis, help maintain training performance loads, and hasten recovery. However, statistical outcomes are mixed and more research is clearly needed before definitive conclusions can be drawn. With this in mind, the following discusses the impact of ingesting this energy/food bar before, during, and following intense exercise on primary and secondary outcomes.

### Primary outcome – glucose homeostasis

Results of this study found that the glycemic and insulinemic response of ingesting the food bar before, during, and following intense exercise was more favorable in maintaining euglycemia than ingesting equivalent amounts of reference carbohydrate (dextrose) as recommended. In this regard, blood glucose levels never increased outside of normal values after FB ingestion compared to an increase of up to 58% with dextrose. Blood glucose levels were significantly higher than baseline before and following exercise in the CHO treatment. Additionally, pre-exercise blood glucose levels in the CHO treatment were significantly higher than FB blood glucose values. Interestingly, even though glucose levels were only modestly increased following FB ingestion, insulin concentration and the GIR were significantly higher than baseline values in both treatments and the GIR following exercise was significantly higher with FB ingestion compared to the dextrose comparator. These findings indicate that FB ingestion promoted a more favorable glucose homeostasis and anti-catabolic hormonal environment. These results support our initial findings that ingestion of this FB promotes a mild increase in blood glucose while serving to increase insulin levels to a greater degree than dextrose [15]. It also provides rationale as to why consumption of this FB may lessen exercise-induced catabolism and/or promote recovery from intense exercise.

There are several possible reasons for these findings. First, amino acid ingestion has been reported to modestly increase insulin levels [35–37] and co-ingestion of protein or amino acids with carbohydrate has been reported to promote a greater effect on insulin [35, 36, 38–42]. The FB studied contained 25 g of IMO with 20 g of whey protein. Thus, it is possible that co-ingestion of IMO and whey protein promoted a greater increase in insulin than the dextrose comparator. Second, the FB was high in fiber and only contained 4 g of digestible carbohydrate (sugar) which would have likely promoted a more gradual release of glucose into the blood thereby facilitating a more sustained increase in insulin. There is evidence that consuming whey protein with fiber affects the glycemic response of co-ingested carbohydrates [43–45]. Therefore, it is possible that co-ingesting whey protein with a high fiber carbohydrate may have augmented insulin response. Third, although IMO is a prebiotic, it is classified as a type of oligosaccharide that has been reported to stimulate growth of “friendly” bacteria which improve gut function through the promotion of activity of the probiotic gut flora [10, 46–48]. While this adaptation would not be expected from acute ingestion, it is possible that intestinal absorption of glucose when provided as IMO may be different from dextrose and/or co-ingestion of IMO with protein may influence glucose uptake differently thereby serving to maintain blood glucose levels to a greater degree while still stimulating insulin responses. While this is speculative, additional research should examine the potential mechanisms associated with these findings as well as whether chronic consumption of IMO may additional benefits.

### Secondary outcomes – Exercise Performance & Recovery

Since we previously found that ingesting this FB promoted a modest and more sustained increase in blood glucose, we hypothesized that ingesting this FB before and during intense exercise may help athletes maintain performance over time. Results of this study provide some support for this hypothesis. In this regard, no significant interactions were observed from GLM analysis. However, analysis of mean changes from baseline with 95% CI’s revealed that leg press and total lifting volume from Set 1 to Set 2 and Set 3 was maintained during the FB treatment (i.e., the means and 95% CI’s crossed baseline values) while significantly decreasing below baseline values with CHO treatment (i.e., the means and 95% CI’s were completely below baseline values). While it is understandable that athletes/experienced lifters may not be able to maintain 70% of 1RM for each exercise during an intense workout due to fatigue, this finding provides some evidence that ingestion of the FB helped maintain the quality of the resistance-exercise training session. Similarly, we found that no significant interactions were observed from GLM analysis. However, analysis of mean changes from baseline with 95% CI revealed that agility performance time was significantly improved from Sprint 1 to Sprint 2 in the FB treatment (i.e., mean changes and 95% CI’s were completely below baseline values) while being unchanged in the CHO treatment (i.e., the means and 95% CI’s crossed baseline values). Moreover, participants performed the first 40-yard sprint − 0.15 s faster with FB compared to CHO. While this latter finding was not statistically significant, it represents a meaningful performance difference from an applied standpoint. These findings provide some evidence that ingesting a FB with a low GI may help athletes sustain high intensity exercise performance to greater degree than the standard practice of consuming carbohydrate alone. Whether this was due to greater digestion time, satiety, and or other factors remains to be determined. However, since we did not observe a significant interaction effect from GLM analysis and only observed differences between treatments from mean change analysis, more research is needed to substantiate this finding.

We also hypothesized that since the FB we previously investigated increased insulin to a greater degree than dextrose and insulin serves as an anticatabolic hormone, ingesting this FB around an intense exercise bout may lessen exercise-induced catabolism and/or perceptions of delayed onset of muscle soreness (DOMS) [1–3, 5]. While there was some evidence that FB ingestion promoted a greater increase in insulin and may lessen perceptions of muscle soreness, it had limited effects on markers of catabolism or inflammation. In this regard, no significant interaction effects were observed in ratings of pain. However, assessment of mean changes from baseline with 95% CI’s revealed that participants rated the pain response to a standard amount of pressure applied to several locations on the thigh to be significantly higher than baseline values after exercise (VM site) and after 48 h of recovery (DVL and MVL) with CHO treatment while ratings in the FB treatment were unchanged from baseline (i.e., means and 95% CI’s crossed baseline). One-way ANOVA analysis revealed that ratings at the VM site also tended to be lower in the FB treatment compared to CHO after exercise. Additionally, participants did not respond as positively to the statement *“I have little muscle soreness”*. These findings support prior reports that whey protein supplementation can affect recovery and/or perceptions of muscle soreness in response to intense training [49–51]. The etiology of this potential benefit remains to be determined but could be related to greater protein synthesis with whey protein ingestion thereby hastening recovery and/or lessening perceptions of pain. However, we found no significant differences between the CHO and FB treatments on markers of whole body catabolism, muscle enzyme efflux, anabolic and catabolic hormones, or inflammatory markers from GLM or assessment of mean changes from baseline with 95% CI’s. These findings support results of other studies that reported limited to no effects of consuming whey protein before and/or during exercise on markers of catabolism or inflammation [52–54]. Additional research is necessary to explore the impact of consuming whey protein with different forms of carbohydrate on markers of recovery from intense exercise.

Finally, analysis of subjective ratings of symptoms revealed that ingestion of CHO and FB before, during and following exercise were well tolerated and had minimal effects on ratings of hypoglycemia, dizziness, headache, fatigue, and stomach upset. Moreover, while the treatments differed in caloric content and sweetness which could influence perceptions about appetite and/or hunger [55]; ingestion of the FB was associated with a greater increase in feeling of fullness with some evidence of less hunger and greater satisfaction from food ratings. While this was somewhat expected given differences in digestion rates and energy intake between treatments, it was interesting given the carbohydrate content was matched. These differences, however, did result in significant differences between treatments in questions related to readiness to perform. Collectively, these findings indicate that the food bar studied serve as a good low-glycemic food choice for active individuals to consume before, during, and/or following intense exercise training.

### Limitations

There were several limitations to this pilot study that should be noted. First, the dextrose comparator was matched to carbohydrate content (25 g) and was a reference carbohydrate for determining the GI and GL of the food source. However, it was provided as a gel and it was not matched for total calories. Given differences in digestion rates, this could have influenced some of the differences observed in glucose homeostasis, performance and/or subjective ratings. However, we felt it was important for this initial study to compare whether ingesting this FB provided greater benefits compared to the standard recommended practice of only consuming carbohydrate drinks and/or gels prior and during exercise [1, 2]. Additional research is needed to determine if incorporating a non-supplemented control group would add to the interpretation of results and/or whether matching total energy intake or using other sources of protein with IMO provides additive benefits. Second, while the study was sufficiently powered and a number of outcome variables were statistically significant, we found borderline significant levels with moderate to large effect sizes suggesting that having a larger n-size may have revealed more significant and consistent findings between GLM and mean change analysis with 95% CI’s. Third, since we evaluated well-trained individuals performing intense exercise, results may not translate to untrained or less fit populations. Fourth, given we were trying to assess a normal training bout of exercise, we limited venous blood assessment data points and therefore may have missed some of the effects of the nutritional interventions on blood markers. Finally, we chose to have participants record and replicate nutritional intake during each treatment and asked them to participate in similar exercise training prior each treatment session. While there were no significant differences in dietary records and participants fasted and refrained from exercise training and NSAID use before reporting to the lab, it is possible that differences in diet, hydration, and/or rest between treatments may have influenced results. Finally, since this study was conducted in an open label manner, it is possible that individual preferences to ingesting the CHO gel and/or food bar may have affected subjective ratings. With that said, the major strengths of this study were the randomized and crossover experimental design and assessment of a typical intense training bout used in the strength and conditioning of athletes. Additionally, the practical assessment of whether having athletes ingest an energy/food bar before, during, and/or following exercise has any influence on glucose homeostasis, exercise training performance, and/or recovery in comparison to the recommended practice of ingesting carbohydrate containing drinks or gels alone.

## Conclusion

Results of this pilot study demonstrated that ingestion of a whey protein with IMO as the source of carbohydrate before, during, and following intense resistance-exercise and sprint conditioning maintained blood glucose and increased insulin to a greater degree than consuming a carbohydrate matched dextrose comparator. Additionally, while GLM analysis revealed no significant interaction effects in performance variables, there was some evidence from analyzing mean changes from baseline with 95% CI’s that FB ingestion helped maintain resistance and sprint exercise performance. However, markers of catabolism and inflammation were not affected. Nevertheless, due to the better glucose response observed, results indicate that this FB can serve as a good low glycemic food option for individuals to take before, during, and/or following intense exercise. Moreover, that this FB may serve as a good low-glycemic food option for pre-diabetic and diabetic populations. Additional research should evaluate the potential benefits of using IMO as a carbohydrate source for active individuals as well as the long-term potential health benefits in functional foods in healthy active, pre-diabetic, and diabetic populations.

## Additional file

Additional file 1: **Table S1.** Energy and macronutrient intake, **Table S2.** Resistance exercise lifting volume, **Table S3.** Sprint performance, **Table S4.** Isokinetic testing results, **Table S5.** Perception of quadriceps muscle soreness, **Table S6.** Markers of catabolism, **Table S7.** Stress and sex hormone response, **Table S8.** Inflammatory marker panel, **Table S9.** Symptoms of hypoglycemia inventory, **Table S10.** Appetite and eating satisfaction inventory, **Table S11.** Readiness to perform questionnaire. (XLSX 39 kb)

## Data Availability

Data and/or statistical analyses are available upon request on a case by case basis for non-commercial scientific inquiry and/or educational use as long as IRB restrictions and research agreement terms are not violated.

## Abbreviations

1RM: One repetition maximum
BF%: Body fat percentage
BMI: Body mass index
CHO: Carbohydrate
CI: Confidence interval
CONSORT: Consolidated Standards of Reporting Trials
CV: Coefficient of variation
DOMS: Delayed onset of muscle soreness
DVL: 25% distal vastus lateralis
DXA: Dual-energy x-ray absorptiometer
FB: Food bar
FYD: 40-yard dash
GI: Glycemic index
GLM: General linear model
GPRS: Graded visual analog scale
IGR: insulin to glucose ratio
IL: Interleukin
IMO: Isomalto-oligosaccharides
MCV: Maximal voluntary contraction
MVL: Midpoint vastus lateralis
NAD: Nebraska agility drill
NSAID: Non-steroidal anti-inflammatory drug
TNF-α: Tumor necrosis factor-α
VM: Vastus medialis
η2: Partial eta-squared
